# Supplementary material for: Time-varying effect in older patients with early-stage breast cancer: a model considering the competing risks based on a time scale
Source: Front Oncol. 2024 Jul 2;14:1352111. doi: 10.3389/fonc.2024.1352111 (PMC11249566; doi:10.3389/fonc.2024.1352111)
Supplement: Supplementary file 1 [file DataSheet_1.docx]

**Supplementary File 1:** data collection and variable

In this study, we extracted data from the SEER database for older patients with early-stage breast cancer.

Patients were included if they met the following criteria: 1. Sex: female; 2. Age: $\geq65$; 3. Diagnosis: breast cancer in 2000-2015; 4. TNM stage: T_1-2_N_1-3_M_0_; and 5. Breast surgery: mastectomy or breast-conserving surgery (BCS). At the same time, several patients were excluded if they met the following exclusion criteria: 1. the covariates used in the model contained missing data; 2. multifocal tumors; and 3. the source of information was an autopsy or a death report only.

Covariates included race, age, marriage, T stage, N stage, histological grade, estrogen receptor (ER) status, progesterone receptor (PR) status, breast surgery, axillary surgery, chemotherapy, and radiotherapy.

Regarding axillary surgery, we defined the removal of 10 or more lymph nodes as axillary lymph node dissection (ALND) and the removal of 1–9 lymph nodes as sentinel lymph node biopsy (SLNB) according to the National Comprehensive Cancer Network (NCCN) guidelines. The event of interest was death from breast cancer. The competing event was death from non-breast cancer.

In this study, we also developed a prediction model for older patients with early-stage breast cancer based on dynamic effect RMTL regression using 3892 patients diagnosed from 2000 to 2012 as a training set and another 1561 patients diagnosed from 2013 to 2015 as an externally validated set.


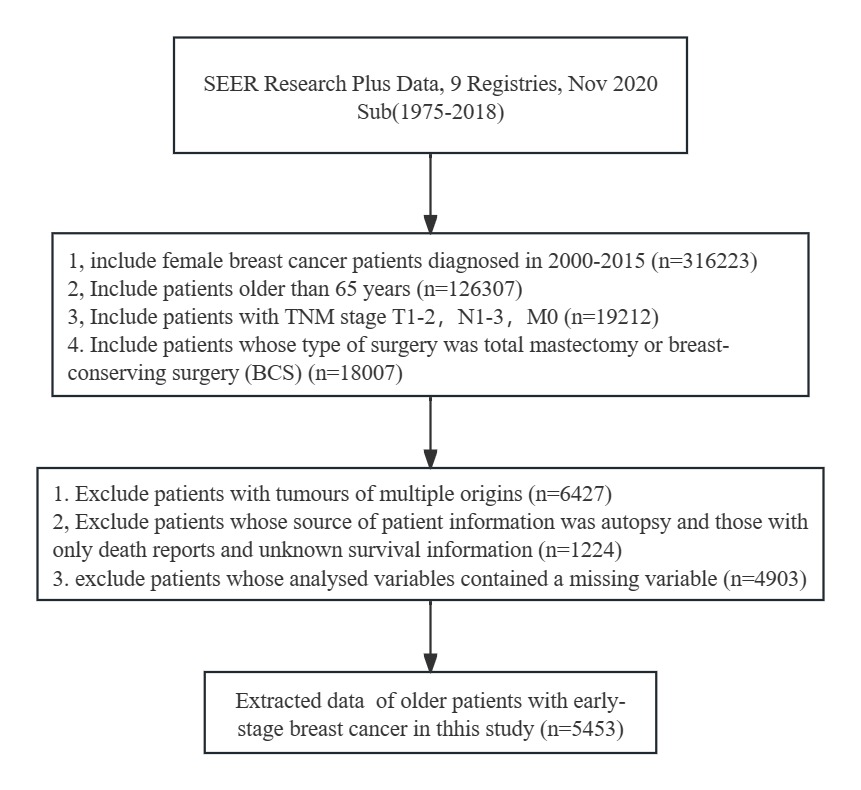


**Supplementary Figure 1** patient selection diagram

**Supplementary Table 1:** Baseline characteristics (number (%)) of the total dataset, training set, and validation set, as well as the chi-square test between the training and validation sets.

| variable | Total data set（N=5454） | Training set（N=3893） | Validation set（N=1561） | $\chi^{2}$ | P value |
| --- | --- | --- | --- | --- | --- |
| Race (%) |  |  |  |  |  |
| white | 4436(81.3) | 3161(81.2) | 1275(81.7) | 0.163 | 0.933 |
| black | 532(9.8) | 383(9.8) | 149(9.5) |  |  |
| other | 485(8.9) | 348(9.0) | 137(8.8) |  |  |
| Age (%) |  |  |  |  |  |
| 65-74 | 3538(64.9) | 2431(62.4) | 1107(70.9) | 34.582 | <0.001 |
| ≥75 | 1915(35.1) | 1461(37.6) | 454(29.1) |  |  |
| Marry (%) |  |  |  |  |  |
| married | 2824(51.8) | 1985(51.0) | 839(53.7) | 3.255 | 0.071 |
| other | 2629(48.2) | 1907(49.0) | 722(46.3) |  |  |
| T stage (%) |  |  |  |  |  |
| T1 | 2767(50.7) | 2022(52.0) | 745(47.7) | 7.796 | 0.005 |
| T2 | 2686(49.3) | 1870(48.0) | 816(52.3) |  |  |
| N stage (%) |  |  |  |  |  |
| N1 | 4120(75.6) | 2861(73.5) | 1259(80.7) | 30.878 | <0.001 |
| N2 | 921(16.9) | 710(18.2) | 211(13.5) |  |  |
| N3 | 412(7.6) | 321(8.2) | 91(5.8) |  |  |
| Grade (%) |  |  |  |  |  |
| Grade Ⅰ | 995(18.2) | 695(17.9) | 300(19.2) | 6.983 | 0.030 |
| Grade Ⅱ | 2505(45.9) | 1761(45.2) | 744(47.7) |  |  |
| Grade Ⅲ&Ⅳ | 1953(35.8) | 1436(36.9) | 517(33.1) |  |  |
| ER status (%) |  |  |  |  |  |
| negative | 894(16.4) | 685(17.6) | 209(13.4) | 14.110 | <0.001 |
| positive | 4559(83.6) | 3207(82.4) | 1352(86.6) |  |  |
| PR status (%) |  |  |  |  |  |
| negative | 1550(28.4) | 1182(30.4) | 368(23.6) | 24.955 | <0.001 |
| positive | 3903(71.6) | 2710(69.6) | 1193(76.4) |  |  |
| Breast surgery (%) | | |  |  |  |
| mastectomy | 1545(28.3) | 1144(29.4) | 401(25.7) | 7.350 | 0.007 |
| BCS | 3908(71.7) | 2748(70.6) | 1160(74.3) |  |  |
| Axillary surgery (%) | | |  |  |  |
| ALND | 2669(48.9) | 1646(42.3) | 1023(65.5) | 239.940 | <0.001 |
| SLNB | 2784(51.1) | 2246(57.7) | 538(34.5) |  |  |
| Chemotherapy(%) | |  |  |  |  |
| No | 2568(47.1) | 1817(46.7) | 751(48.1) | 0.851 | 0.356 |
| Yes | 2885(52.9) | 2075(53.3) | 810(51.9) |  |  |
| Radiation (%) |  |  |  |  |  |
| No | 129(2.4) | 90(2.3) | 39(2.5) | 0.096 | 0.757 |
| Yes | 5324(97.6) | 3802(97.7) | 1522(97.5) |  |  |

**Supplementary Table 2:** Regression coefficients of dynamic-effect RMTL regression (all data)

| Variable | Time  function | Coefficient | SE | Z value | P value |
| --- | --- | --- | --- | --- | --- |
| Intercept | $1$ | -0.190 | 0.071 | -2.669 | 0.008 |
|  | $l$ | 0.114 | 0.024 | 4.830 | <0.001 |
| Age (ref: 65-74) |  |  |  |  |  |
| age 75+ | $1$ | -0.095 | 0.033 | -2.827 | 0.005 |
|  | $l$ | 0.044 | 0.010 | 4.266 | <0.001 |
| T stage (ref: T1) |  |  |  |  |  |
| T2 | $l^{2}$ | 0.006 | 0.001 | 8.050 | <0.001 |
| N stage (ref: N1) |  |  |  |  |  |
| N2 | $l^{2}$ | 0.006 | 0.001 | 5.105 | <0.001 |
| N3 | $1$ | -0.414 | 0.082 | -5.080 | <0.001 |
|  | $l$ | 0.168 | 0.025 | 6.792 | <0.001 |
| Grade (ref: grade Ⅰ) |  |  |  |  |  |
| Ⅱ | $l^{2}$ | 0.002 | 0.001 | 2.915 | 0.004 |
| Ⅲ & Ⅳ | $1$ | -0.211 | 0.041 | -5.197 | <0.001 |
|  | $l$ | 0.085 | 0.013 | 6.591 | <0.001 |
| ER status (ref: negative) |  |  |  |  |  |
| positive | $1$ | 0.253 | 0.057 | 4.447 | <0.001 |
|  | $l$ | -0.121 | 0.021 | -5.664 | <0.001 |
|  | $l^{2}$ | 0.005 | 0.001 | 4.111 | <0.001 |
| PR status (ref: negative) |  |  |  |  |  |
| positive | $l$ | -0.025 | 0.009 | -2.938 | 0.003 |
| Breast surgery (ref: mastectomy) |  |  |  |  |  |
| BCS | $l^{2}$ | -0.002 | 0.001 | -2.424 | 0.015 |
| Chemotherapy (ref: no) |  |  |  |  |  |
| yes | $1$ | 0.147 | 0.041 | 3.563 | <0.001 |
|  | $l$ | -0.084 | 0.016 | -5.242 | <0.001 |
|  | $l^{2}$ | 0.004 | 0.001 | 3.399 | 0.001 |
