# Supplementary material for: Time-varying effect in older patients with early-stage breast cancer: a model considering the competing risks based on a time scale
Source: Front Oncol. 2024 Jul 2;14:1352111. doi: 10.3389/fonc.2024.1352111 (PMC11249566; doi:10.3389/fonc.2024.1352111)
Supplement: Supplementary file 2 [file Table_2.docx]

**Supplementary Table 2:** Regression coefficients of dynamic-effect RMTL regression (all data)

| Variable | Time  function | Coefficient | SE | Z value | P value |
| --- | --- | --- | --- | --- | --- |
| Intercept | $1$ | -0.190 | 0.071 | -2.669 | 0.008 |
|  | $l$ | 0.114 | 0.024 | 4.830 | <0.001 |
| Age (ref: 65-74) |  |  |  |  |  |
| age 75+ | $1$ | -0.095 | 0.033 | -2.827 | 0.005 |
|  | $l$ | 0.044 | 0.010 | 4.266 | <0.001 |
| T stage (ref: T1) |  |  |  |  |  |
| T2 | $l^{2}$ | 0.006 | 0.001 | 8.050 | <0.001 |
| N stage (ref: N1) |  |  |  |  |  |
| N2 | $l^{2}$ | 0.006 | 0.001 | 5.105 | <0.001 |
| N3 | $1$ | -0.414 | 0.082 | -5.080 | <0.001 |
|  | $l$ | 0.168 | 0.025 | 6.792 | <0.001 |
| Grade (ref: grade Ⅰ) |  |  |  |  |  |
| Ⅱ | $l^{2}$ | 0.002 | 0.001 | 2.915 | 0.004 |
| Ⅲ & Ⅳ | $1$ | -0.211 | 0.041 | -5.197 | <0.001 |
|  | $l$ | 0.085 | 0.013 | 6.591 | <0.001 |
| ER status (ref: negative) |  |  |  |  |  |
| positive | $1$ | 0.253 | 0.057 | 4.447 | <0.001 |
|  | $l$ | -0.121 | 0.021 | -5.664 | <0.001 |
|  | $l^{2}$ | 0.005 | 0.001 | 4.111 | <0.001 |
| PR status (ref: negative) |  |  |  |  |  |
| positive | $l$ | -0.025 | 0.009 | -2.938 | 0.003 |
| Breast surgery (ref: mastectomy) |  |  |  |  |  |
| BCS | $l^{2}$ | -0.002 | 0.001 | -2.424 | 0.015 |
| Chemotherapy (ref: no) |  |  |  |  |  |
| yes | $1$ | 0.147 | 0.041 | 3.563 | <0.001 |
|  | $l$ | -0.084 | 0.016 | -5.242 | <0.001 |
|  | $l^{2}$ | 0.004 | 0.001 | 3.399 | 0.001 |
